# Long-Running Comparison of Feed-Water Scaling in Membrane Distillation

**DOI:** 10.3390/membranes10080173

**Published:** 2020-07-31

**Authors:** Mohammad Rezaei, Albraa Alsaati, David M. Warsinger, Florian Hell, Wolfgang M. Samhaber

**Affiliations:** 1 Institute of Process Engineering, Johannes Kepler University Linz, Altenberger Strasse 69, 4040 Linz, Austria; wolfgang.samhaber@jku.at; 2 Birck Nanotechnology Center, School of Mechanical Engineering, Purdue University, West Lafayette, IN 47907, USA; aalsaati@purdue.edu (A.A.); dwarsing@purdue.edu (D.M.W.); 3 VA TECH WABAG GmbH, Dresdner Strasse 89-91, 1200 Vienna, Austria; Florian.Hell@wabag.com

**Keywords:** vacuum membrane distillation, long-term performance tests, reverse osmosis brine, scaling, wetting, antiscalant

## Abstract

Membrane distillation (MD) has shown promise for concentrating a wide variety of brines, but the knowledge is limited on how different brines impact salt scaling, flux decline, and subsequent wetting. Furthermore, past studies have lacked critical details and analysis to enable a physical understanding, including the length of experiments, the inclusion of salt kinetics, impact of antiscalants, and variability between feed-water types. To address this gap, we examined the system performance, water recovery, scale formation, and saturation index of a lab-scale vacuum membrane distillation (VMD) in long-running test runs approaching 200 h. The tests provided a comparison of a variety of relevant feed solutions, including a synthetic seawater reverse osmosis brine with a salinity of 8.0 g/L, tap water, and NaCl, and included an antiscalant. Saturation modeling indicated that calcite and aragonite were the main foulants contributing to permeate flux reduction. The longer operation times than typical studies revealed several insights. First, scaling could reduce permeate flux dramatically, seen here as 49% for the synthetic brine, when reaching a high recovery ratio of 91%. Second, salt crystallization on the membrane surface could have a long-delayed but subsequently significant impact, as the permeate flux experienced a precipitous decline only after 72 h of continuous operation. Several scaling-resistant impacts were observed as well. Although use of an antiscalant did not reduce the decrease in flux, it extended membrane operational time before surface foulants caused membrane wetting. Additionally, numerous calcium, magnesium, and carbonate salts, as well as silica, reached very high saturation indices (>1). Despite this, scaling without wetting was often observed, and scaling was consistently reversible and easily washed. Under heavy scaling conditions, many areas lacked deposits, which enabled continued operation; existing MD performance models lack this effect by assuming uniform layers. This work implies that longer times are needed for MD fouling experiments, and provides further scaling-resistant evidence for MD.

## 1. Introduction

The most commonly used process for water desalination is reverse osmosis (RO), primarily due to its comparatively low energy consumption [1,2]. However, in practice, RO maximum recovery is limited to 35–60% since a higher recovery ratio would require overcoming osmotic pressures above 80 bar, which is neither economical nor mechanically feasible for most membranes [3,4,5]. Further, recent studies have pointed out the environmental harm of disposed RO concentrates (pollution by residual chlorine and heavy metals [6,7], which is particularly problematic for marine plants [8]).

On the other hand, membrane distillation utilizes thermal energy to provide the driving pressure force, which does not deteriorate significantly for high salinity feed [9,10,11,12,13,14,15]. Additionally, supplementing the membrane distillation (MD) module with a vacuum pump on the distillate side, commonly known as vacuum membrane distillation (VMD), further improves the performance for extremely high salinity feed [16,17,18,19]. Hence, the RO process can potentially achieve a relatively high recovery rate by integrating a VMD unit to further process the brine [20].

Numerous studies have analyzed the feasibility of RO-MD processes, and several have shown that pure water and salt deposits can be obtained from concentrated feeds, with water recovery in the range of 20–60% [21,22,23,24,25]. Most recently, Zou et al. [26] studied the concentration of seawater reverse osmosis brine by means of a submerged VMD process. They concluded that, although the effect of some inorganic compounds could be eliminated by a pretreatment process, an adequate method for reducing membrane fouling remains to be found. With this in mind, a deep understanding of the fouling mechanism and the effect of RO chemical additives in extended operation is crucial for implementing VMD technology into industrial applications.

In fact, several studies investigated the long-term operation of membrane distillation technology [27,28,29,30]. However, those investigations focused on a low fouling feed solution in a direct-contact membrane distillation (DCMD) configuration and analyzed the change in surface morphology of the membrane. Yet, for high fouling application, the presence of fouling and scaling obstruct the path to the membrane by forming a layer on the membrane surface [31,32,33]. As a result, understanding the mechanism of fouling and crystallization is more important in high fouling application compared to the membrane surface morphology effect. Thus, there remains a need for long-term studies that investigate fouling and wetting in the VMD process in depth. Moreover, for high-concentration feeds, the antifouling and antiscaling techniques must be investigated in more detail to analyze their effectiveness in long-term operation.

The objective of this work was to determine the practicability of concentrating a variety of water types in a lab-scale VMD apparatus to achieve a high water-recovery ratio in long-term performance tests. Additionally, we investigated the limitations of the process due to scaling. In phase I of this investigation, the membrane was characterized using low-salinity feeds (Linz tap water) and an aqueous sodium chloride solution. In phase II, water recovery ratio and flux reduction due to scale formation of a synthetic RO brine were compared to the results for the low-salinity feeds. Finally, in phase III, the effect of using an antiscalant on permeate flux was analyzed.

## 2. Materials and Methods

### 2.1. Laboratory Apparatus

**Figure 1:**
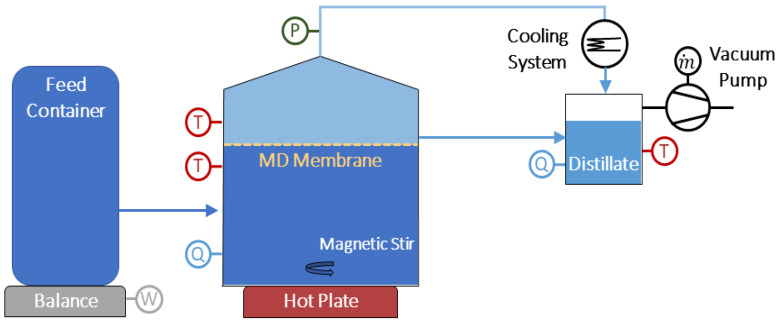
Vacuum membrane distillation (VMD) process schematic. The setup consisted of a feed container, a stirred membrane cell, a distillate collection vessel, a feed container, a magnetic stirrer, an external condenser, a balance, and a vacuum pump.

The experiments were performed with a lab-scale VMD apparatus (Figure 1) using a flat sheet membrane with an area of 50 cm^2^. The laboratory apparatus consisted of a feed container, a stirred membrane cell, a distillate collection vessel (5000 mL, Glass, Gaßner Glastechnik, Munich, Germany), a magnetic stirrer (IKA ika_rh_basic_digital, Staufen, Germany), an external condenser (Liebig cooler, Gaßner Glastechnik, Munich, Germany), a platform balance (Kern KXS_TM-BA_IA-d-1310, Balingen, Germany), and a vacuum pump (M-0160 KNF Neuberger miniport, Freiburg im Breisgau, Germany), as illustrated in Figure 1. We connected the feed solution to the membrane test cell, which filled the 300 mL volume of the feed side of the cell. The solution in the cell was heated and stirred at a rate of 750 rpm using the magnetic stirrer. The vacuum pump provided a pressure difference, causing water vapor to flow through the hydrophobic membrane pores to an external condenser, where the permeate was collected. New feed solution from the feed container compensated for the water evaporated in the cell. We determined the distillate flow by weighing the reduced brine mass in the feed reservoir. Temperature and conductivity measurements were made both in the membrane test cell and on the permeate side of the membrane. Feed temperature, permeate temperature, and gauge pressure were kept constant throughout the experiments at 75 °C, 21 °C, and −30 mbar, respectively.

Two critical processing parameters in this context are concentration factor (*f*) and membrane water recovery. The concentration factor was calculated as [34]:

(1) $$f=M_{C}/M_{F},$$

where $M_{C}$ is the feed mass under the membrane in the cell and $M_{F}$ is the sum of $M_{C}$ and the mass of liquid dispensed from the feed tank. Water recovery (*WR*) was calculated as:

(2) $$WR=100(M_{F}-M_{C})/M_{F}=100\left(1-1/f\right).$$

All experiments were repeated multiple times using new membranes in each experiment. In all cases, the experiments were stopped once the permeate flux had dropped by at least 50%. Several chemical and physical analyses were conducted to examine the compositions of feed, distillates, and final MD brines.

### 2.2. Membrane Material

A commercial hydrophobic QL833 AspireTM Microfiltration membrane purchased from General Electric was used. The membrane uses polytetrafluoroethylene (PTFE) as a functional layer and polypropylene (PP) as backer material with a reference pore size of 0.2 µm and a thickness of 0.12–0.2 mm. Membrane porosity and water entry pressure are reported to be 88% [35] and 3.5 bar [36] (ASTMD751), respectively.

### 2.3. Feed Waters

Four types of MD feed were used in this work: Linz tap water, NaCl solution (5.25 g/L), a synthetic RO brine, and the synthetic RO brine with “Genesys LF” antiscalant. The tap water had an electrical conductivity of 663 µS/cm, a pH of 7.75, and a bicarbonate concentration of 250 mg/L. Since the tap water was used to prepare the synthetic RO brine, its composition was taken into account when adding substances to obtain the proposed brine composition (Table 1). The synthetic feed was immediately analyzed to check its composition. The final composition of the synthetic RO brine is given in Table 2.

### 2.4. Salt Saturation Modeling

Aqueous speciation equilibrium analysis was performed by means of PHREEQC [37] to identify the distribution of aqueous species and corresponding saturation indices based on the chemical analysis in Table 2. The results showed good agreement between the target and the solutions obtained. The final salinity of synthetic RO brine was 8.0 g/L according to measurement with a Mettler Toledo infrared dryer.

### 2.5. Antiscalant

Genesys antiscalant contains an aqueous solution of neutralized phosphonate, including phosphonic acid, (nitrilotris(methylene)) tri-, sodium salt 20–50%. The Genesys LF antiscalant inhibits (i) calcium sulfate, barium sulfate, strontium sulfate and calcium fluoride scales and (ii) iron, silica, and aluminum fouling [38]. The recommended doses for recovery ratios of 75% and 85% are 2.5 mg/L and 4 mg/L, respectively. According to the manufacturer, the antiscalant can be diluted, but should always be used fresh. The synthetic feed with antiscalant was prepared by adding 1.5 mL of the diluted antiscalant at a dilution ratio of 1:100 to a 5 L synthetic feed volume.

## 3. Results

### 3.1. Tap-Water-Based VMD Results

**Figure 2:**
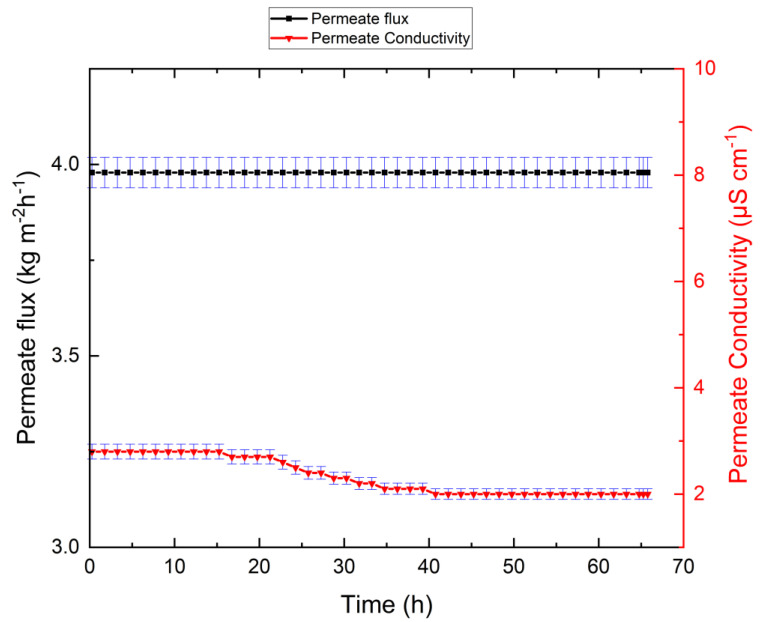
Linz tap water VMD test showing permeate flux and permeate electrical conductivity against time. The system exhibited no reduction in permeate flux during the experiment.

A preliminary series of experiments was designed to test the behavior of the membrane with low-salinity feed. For this purpose, Linz tap water was used as feed, and permeate electrical conductivity and permeate flux were measured for 65 h until a recovery ratio of 80% was achieved. Figure 2 shows the VMD performance for the Linz tap water feed.

As illustrated in Figure 2, the system exhibited no reduction in permeate flux throughout the experiment. The permeate flux was measured to be 3.98 kg m^−2^ h^−1^ and remained relatively constant until the end of the experiment. Interestingly, the permeate electrical conductivity decreased from its initial value of 2.8 to 2.0 µS/cm during the experiment. The slight decrease of permeate electrical conductivity is due to the collection of pure water and dilution of existing permeate in the distilled tank. This low level of permeate electrical conductivity indicates the absence of wetting.

### 3.2. VMD Results for Synthetic Brine Without Antiscalant

**Figure 3:**
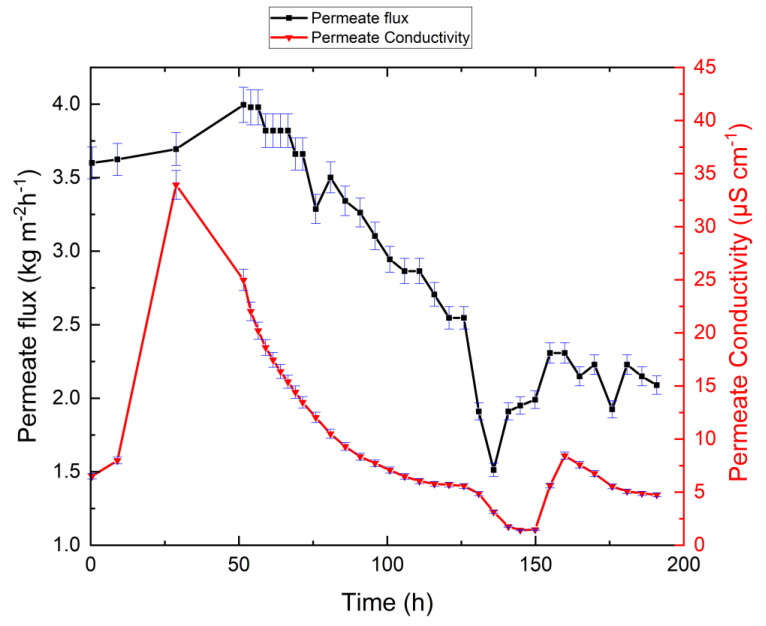
Permeate flux and permeate electrical conductivity as functions of time for the synthetic feed system without antiscalant. The permeate flux decreased, which indicates fouling, but permeate electrical conductivity remained at low levels, which indicates the absence of wetting. Feed temperature, permeate temperature, and gauge pressure were kept constant at 75 °C, 21 °C, and −30 mbar during the experiments, respectively.

The experiments with synthetic RO brine feed without antiscalant were carried out to assess system performance for the highest possible recovery ratio and permeate flux behavior. Figure 3 plots VMD performance as a function of time, and Table 3 shows the chemical analysis of the retentate obtained at the end of the experiment. As illustrated in Figure 3, the permeate flux initially reached 4.0 kg m^−2^ h^−1^, and after 72 h it gradually decreased to 1.5 kg m^−2^ h^−1^. The permeate electrical conductivity increased to 44.8 µS/cm within the first 30 h of the experiment, then decreased to 1.5 µS/cm and reached 5.1 µS/cm at the end of the experiment. The increase in permeate electrical conductivity can be explained as follows: as shown in Table 2, the synthetic RO brine feed contains several dissolved ions, including sulfate, bicarbonate, calcium, chloride, potassium, sodium, and magnesium. The feed is under atmospheric pressure and is sucked into the testing cell due to evaporation. As the distillate side is under vacuum, CO_2_ equilibrates accordingly. When the feed is heated, the bicarbonate ions shift to carbonate, water, and carbon dioxide [39]. This leads to the release of carbon dioxide and its passage through the membrane pores, which causes the permeate electrical conductivity to increase and, consequently, alkaline scaling on the membrane surface (Figure 3). The increase of retentate pH value in Table 3 shows this effect. This condition has also been reported by other researchers [40]. The overall reactions describing the crystallization of the CaCO_3_ scale are represented by:

(3) $$CaCl_{2}\to Ca^{2+}+2Cl^{-}$$

(4) $$2HCO_{3}^{-}\to CO_{2}↑+H_{2}O+CO_{3}^{2-}$$

(5) $$Ca^{2+}+2HCO_{3}^{-}\to CaCO_{3}↓+CO_{2}↑+H_{2}O$$

where the feed temperature of 36 °C is the lowest temperature for the formation of CaCO_3_ [41]. The lower concentrations of calcium, magnesium, barium, strontium, and silica in MD-brine compared to MD-feed presented in Table 3 are a clear indication for precipitation. As illustrated in Figure 3, complete decomposition of $HCO_{3}^{-}$ into $CO_{3}^{2-}$ occurred at 75 °C during the first 30 h of the experiment, when high permeate electrical conductivity was observed. Precipitation of Mg(OH)_2_ started at approximately 74 °C, and the scaling intensity increased with increasing concentration factor. The slight initial increase of permeate flux was three-fold. This is attributed to it reaching a steady-state condition, along with the dissolution of CO_2_ in permeate water, and some surface wettings of pores. This is supported by the increase of permeate electrical conductivity during this period. However, as the operation continues, the permeate flux becomes decreased due to scaling. Note that the permeate flux dataset in Figure 3 was smoothed for better visibility.

**Figure 4:**
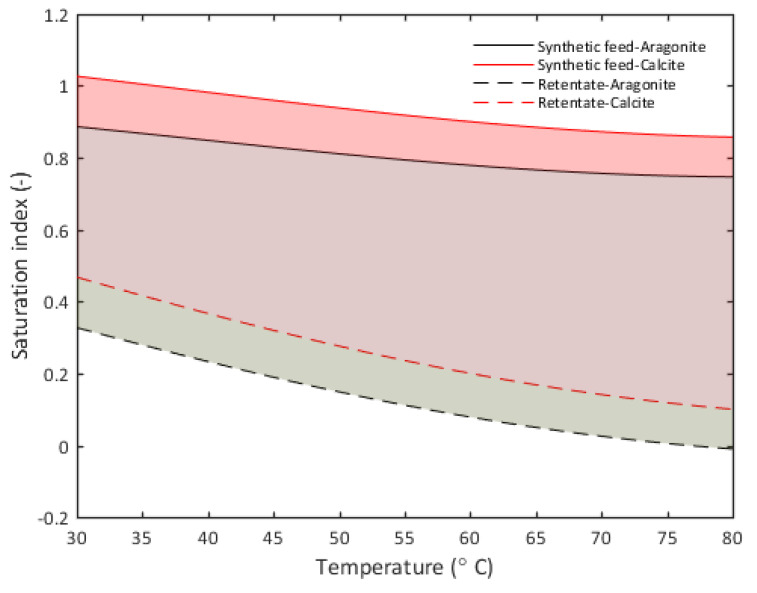
Aqueous speciation equilibrium of synthetic RO brine feed without antiscalant and its corresponding retentate. Solid lines indicate the saturation indices of the main mineral precipitates in synthetic RO brine feed without antiscalant, while dashed lines indicate the corresponding saturation indices for the retentate. The shaded area indicates the drop in saturation indices caused by precipitation on the membrane surface. Modeled using PHREEQC software.

The alkaline scaling mechanism depends primarily on the temperature, the concentration of the brine, and the hydraulic conditions in the test cell [42]. Additional parameters including heat transfer rate, the state of the heat transfer surface, the degree of supersaturation, and the rate of CO_2_ evolution also play a role [43]. The solubility of many salts increases with rising temperature, and they crystallize on the membrane surface only when their concentrations are high. Scale precipitates are formed by those salts whose solubility is limited and decreases with increasing temperatures. For instance, the solubility of a calcium carbonate crystal is inverse: its solubility in water decreases as the temperature rises [44]. The following equation describes the solubility product of CaCO_3_:

(6) $$log(K_{sp})=-171.9773-0.077993T+\frac{2903.293}{T}+71.595\;log\left(T\right),$$

where *T* is the absolute temperature [*K*], and *K_SP_* is the solubility product of CaCO_3_ in molar units. We found similar solubility trends in the speciation analysis of feed water shown in Figure 4.

As illustrated in Figure 3, solid-phase deposition did not occur immediately. The induction period of CaCO_3_ nucleation was around 72 h, and it decreased as supersaturation increased. Using the synthetic RO brine as a feed for the MD did therefore not result in a fast reduction in permeate flux. However, salt deposition on the membrane surface occurred after 82% recovery, which resulted in a gradual reduction in the permeate flux. Note that temperature and concentration polarization phenomena can facilitate precipitation of deposits on the membrane [15,45,46,47].

The higher initial permeate conductivity may be due to CO_2_ (resulting from bicarbonate in the water) passing through membrane pores together with the water vapor. One might suspect that the complete wetting of large membrane pores may have occurred, causing an increase in electrical conductivity. However, as shown in Figure 3, after 18 h, the permeate electrical conductivity decreased due to dilution of the distilled permeate flowing into the permeate tank. The initial increase in permeate electrical conductivity has also been reported in the literature [48,49,50].

### 3.3. Saturation Index Analysis

A multi-component aqueous speciation equilibrium model is performed to calculate the distribution of aqueous species and saturation indexes based on water elements concentrations provided in Table 2 and Table 3. A commercial database, PHREEQC, was used for constant equilibrium values, K, of all possible species formed. The calculation accounts for mole balance, the activity of water, ionic strength, and pH value effect. The results from aqueous speciation equilibrium analysis showed that the calcium minerals with the highest saturation indices were aragonite (CaCO_3_), calcite, and dolomite (CaMg(CO_3_)_2_). Among these, dolomite had the highest saturation index in both the synthetic RO brine feed without antiscalant and its corresponding retentate solution. However, studies have shown that temperature has a stronger influence on dolomite precipitation than the saturating index [51]. Similarly, some silica minerals had high saturation indices. According to the literature [52], supersaturated silica does not typically precipitate, but forms colloids suspended in solution. Hence, at temperatures below 100 °C, the predominant mineral precipitates were calcite and aragonite, as shown in Figure 4, where the drop in saturation indices in retentate was caused by precipitation on the membrane surface. The saturation indices of secondary minerals are shown in Appendix A.

### 3.4. Effect of Antiscalant on the System

The effect of antiscalant on system performance was also studied to investigate the possibility of achieving a higher recovery ratio and of extending the time until scaling and the beginning of crystallization on the membrane surface.

**Figure 5:**
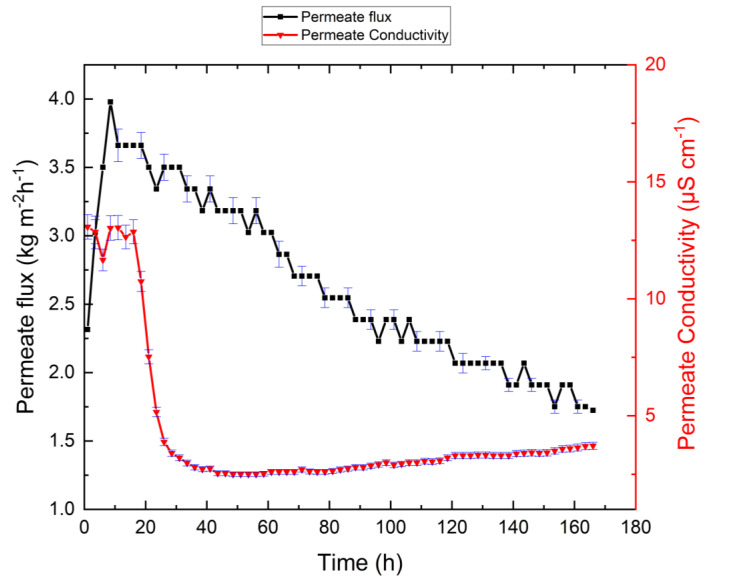
Permeate flux and permeate electrical conductivity as functions of time for the synthetic brine feed system with antiscalant. The antiscalant led to higher initial permeate flux compared to the system without antiscalant. Permeate electrical conductivity remained relatively low.

Figure 5 illustrates the system performance for the synthetic feed treated with the Genesys LF antiscalant. As shown in Figure 5, the initial permeate flux decreased from 3.97 kg m^−2^ h^−1^ to 1.59 kg m^−2^ h^−1^ after 125 h. The initial permeate electrical conductivity was around 15 µS/cm within the first 10 h of the experiment, then decreased to 2.50 µS/cm, and gradually increased to 3.70 at the end of the experiment. The brine electrical conductivity increased linearly from 9.47 mS/cm to 47 mS/cm, while a final recovery ratio of 88% was achieved. Compared to the system without antiscalant, the initial permeate flux was higher, while the permeate electrical conductivity did not increase to more than 15 µS/cm. Further, the antiscalant influenced brine electrical conductivity, and kept it below 47.5 mS/cm, while the recovery ratio remained the same (see Appendix A).

**Figure 6:**
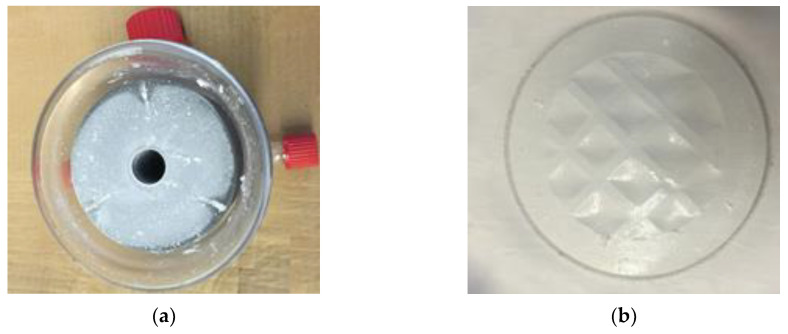
After the experiment with synthetic RO brine: (**a**) Membrane module; (**b**) Membrane surface. Salt crystals deposits are visible, which can easily be removed by washing with water.

Figure 6a,b show the membrane module and the surface of the membrane after the experiment, respectively. As reported by other researchers [53], the scaling on the surface of the membrane was reversible, and washing the membrane surface with distilled water removed the scales efficiently. The washed membrane exhibited more than 95% permeate flux restoration.

### 3.5. Effect of Wetting on Membrane Performance

**Figure 7:**
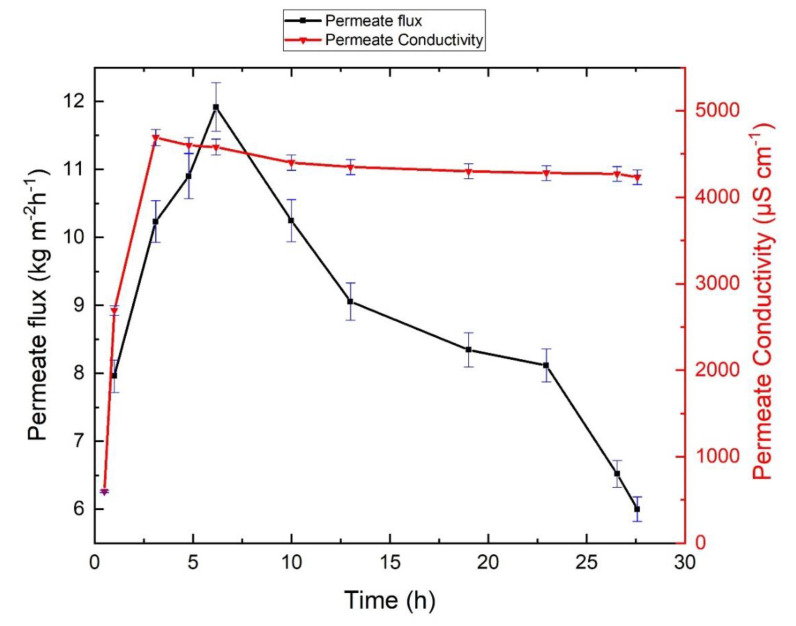
Effect of forced (i.e., high-pressure) wetting on system performance. Operation of the process above liquid entry pressure (LEP) led to rapid passage of liquid feed to the permeate side of the membrane, which drastically increased permeate electrical conductivity.

We also investigated the effect of high vacuum pressure that causes membrane pore wetting on membrane performance. Pore wetting is critical in MD processes because vapor-liquid interfaces are established at the pore entry of the membrane, and the permeate moves only as vapor through the membrane pores [54,55]. Membrane wetting results in passage of liquid feed through the membrane pores, which increases permeate electrical conductivity and lowers separation efficiency [56,57]. Hydrophobic membranes do not allow transfer of liquid through the pores below a particular pressure difference called liquid entry pressure (LEP) [58,59,60]. Figure 7 shows the effect of membrane wetting on system performance when a transmembrane pressure higher than the LEP was applied to the membrane.

Although a permeate flux of 12 kg m^−2^ h^−1^ was achieved, wetting caused permeate electrical conductivity to increase to 4690 µS/cm. High permeate flux and conductivity are signs of liquid passing through the membrane pores. Table 4 shows the chemical analysis of the permeate contaminated by the liquid feed as a result of pore wetting. Evidently, the ions were carried through the membrane pores by liquid feed. This experiment revealed the importance of operating at a vacuum pressure that remains below the liquid penetration pressure.

### 3.6. Effect of Retentate Salinity on Permeate Flux

As can be seen in Figure 3 and Figure 5, permeate flux decreased considerably. Although permeate flux can be reduced by both high salinity and membrane fouling, salinity was not significant in this case. The impact of water vapor pressure at these salinities (<100 g/L) is small according to other authors [61,62,63] and Equation (7) [64,65]:

(7) $$\frac{P_{v,fw}}{P_{v,sw}}=1+0.57357\left[\frac{S_{A}}{1000-S_{A}}\right],$$

where $P_{v,fw}$ is the freshwater vapor pressure (mbar), $P_{v,sw}$ is the saline water vapor pressure (mbar) and $S_{A}$ is the absolute salinity (g/kg). Therefore, we conclude that scaling on the membrane surface caused permeate flux reduction in both the system with and that without antiscalant.

### 3.7. Effect of Scaling on Membrane Performance

A comparison of the feed systems with and without antiscalant revealed a significant impact of scaling on the performance of the membrane. For instance, usage of the antiscalant resulted in a higher average permeate flux and a lower permeate electrical conductivity than in the system without antiscalant. We achieved recovery ratios higher than 88% in both systems. In contrast, the final brine electrical conductivity of the antiscalant-treated system reached a lower value (47.50 mS/cm) than the untreated system (86.60 mS/cm). Furthermore, using antiscalant increased the time until membrane scaling from 28 h to 59 h. Scaling – and consequently wetting—occurred when the amount of salt (i.e., brine and in-situ crystals) in the system reached approximately 680.0 g/L water.

As previously discussed, deposition on the membrane surface caused a reduction in permeate flux, contributed to more wetting, decreased membrane performance, and reduced heat efficiency [66]. Scaling (for the high-concentration brine) created an extra layer on the membrane surface that consisted of particles existing in the liquid. However, some areas of the membrane surface stayed free from visible scaling. The primary salts responsible for scaling were calcium crystals with the smallest solubility (i.e., CaCO_3_ and CaSO_4_) [67]. However, in all experiments, bulk crystallization and unbound cake scaling were reversible, and washing the membrane surface with distilled water removed the scales efficiently.

## 4. Summary

Table 5 compares and contrasts the results obtained for various feed types. As summarized in Table 5, permeate flux and permeate electrical conductivity varied between 1.59 to 3.98 kg m^−2^ h^−1^ and 2.0 to 44.8 µS/cm for various feed types. The highest permeate flux of 3.98 kg m^−2^ h^−1^ with a permeate electrical conductivity of 2 µs/cm was achieved for the system with low-feed salinity (Linz tap water) at a feed temperature of 75 °C and a gauge pressure of −30 mbar. The long-term tests led to average flux reductions by 49% and 50%, corresponding to water recovery ratios of 91% and 88% after 128 h and 148 h of operation for the synthetic brine feed systems with and without antiscalant, respectively.

## 5. Conclusions

The long-running lab-scale VMD experiments for water desalination were performed using a flat sheet hydrophobic (PTFE/PP) membrane to investigate the highest possible water recovery and to examine the effect of scaling on system performance for various feed waters. A series of preliminary experiments were designed to test the behavior of the membrane with a low-salinity feed (Linz tap water) and aqueous sodium chloride solution (5.25 g/L). The study’s performance emphasized the importance of longer duration experiments and demonstrated scaling resistance of MD.

Importantly, our results showed that combining RO and VMD increases water recovery from 40% to 91%, which corresponds to a brine concentration factor of up to 11.1. Note that the application of the synthetic RO brine as feed for VMD did not result in an immediate decrease in permeate flux compared to using low-salinity feed. Rather, permeate flux started to decrease at a water recovery rate of 82% due to deposition of salts on the membrane surface. In addition to high retentate concentrations, we observed scaling in VMD; however, large areas of the membrane remained without visible scaling. Scaling on the membrane surface was caused by the decomposition of bicarbonates present in the brine under feed temperature conditions. Substantial membrane scaling actually reduced the concentration of salts in the retentate. Notably, in all cases, surface scaling could be washed away effectively by cleaning the membrane surface with distilled water.

Furthermore, salt deposits around membrane pores were found to alter membrane hydrophobicity, which led to membrane wetting. The use of Genesys LF antiscalant delayed both flux reduction and membrane wetting. However, it also led to a decrease in the average permeate flux.

There were numerous observations that are inconsistently considered in models of scale, along with their impact on performance. These included scale deposits being patchy, ease of cleaning, ability to scale without wetting in some conditions, and the strong delayed time-dependent behavior of scaling.

Future investigations will consider the effect of stirring on system performance and scaling development and its morphology. Fluid circulations within the membrane module affect the permeate flux significantly. Specifically, high fluid circulation reduces ion residence time on the membrane surface and thus surface crystallization. Similar findings have been reported by other researchers [68,69].

Overall, the experiments show that VMD has the potential to be integrated with RO to achieve higher water recovery ratios.

## Figures and Tables

**Table 1:** Composition of Linz tap water and the substances added to prepare the synthetic reverse osmosis (RO) brine solution.

Substance	Molar Mass	g/m^3^ Tap Water	Added to Tap Water (g/m^3^)	Solution (g/m^3^)
Al_2_(SO_4_)_3_	342.13588	-	2	2
BaCl_2_	208.236	-	0.45	0.45
CaCl_2_	110.9864	285	286	571
FeCl_3_	162.206	-	0.35	0.35
HCL	36.4609	-	750	750
KCl	74.5513	8.3	79.3	87.6
MgCl_2_	95.211	105	95	200
NaCl	58.44277	-	2200	2200
Na_2_CO_3_	105.98874	60	1927	1987
NaF	41.988173	-	4.5	4.5
NaNO_3_	84.99467	31.8	550.2	582
Na_2_SO_4_	142.03714	41.8	1244.2	1286
Na_3_PO_4_	163.94067	-	5.8	5.8
MnCl_2_	125.844	-	0.1	0.1
SrCl_2_	158.526	-	4.7	4.7
Na_2_SiO_3_	122.06324	-	250	250

**Table 2:** Composition of the synthetic RO brine solution.

Physical Parameters	Results	Unit
pH value	8.9	-
electrical conductivity	10,110	µs/cm
**Chemical parameters**		
bicarbonate	858	mg/L
calcium (Ca)	17.4	mg/L
magnesium (Mg)	48.9	mg/L
nitrate	357	mg/L
nitrite	0.03	mg/L
chloride (${\mathrm{Cl}}^{-}$)	2470	mg/L
sulfate	1130	mg/L
iron (Fe)	0.03	mg/L
manganese (Mn)	0.01	mg/L
aluminum (Al)	0.05	mg/L
sodium (Na)	2430	mg/L
potassium (K)	62.7	mg/L
**Inorganic trace constituents**		
orthophosphate	0.35	mg/L
fluoride	2	mg/L
**Elements (metals and semimetals)**		
barium (Ba)	54	µg/L
silicon (calculated as SiO_2_)	40.4	mg/L
strontium (Sr)	862.1	µg/L

**Table 3:** Chemical analysis of the retentate (liquid solution and not including in-situ crystals) obtained after the experiment for the system with synthetic RO brine feed without antiscalant.

Physical Parameters	Results	Unit
pH value	9.12	-
electrical conductivity	81,900	µs/cm
**Chemical parameters**		
bicarbonate	6750	mg/L
calcium (Ca)	1.7	mg/L
magnesium (Mg)	18.2	mg/L
nitrate	4150	mg/L
nitrite	0.7	mg/L
chloride (${\mathrm{Cl}}^{-}$)	28,700	mg/L
sulfate	13,100	mg/L
iron (Fe)	<0.03	mg/L
manganese (Mn)	<0.01	mg/L
aluminum (Al)	<0.05	mg/L
sodium (Na)	28,900	mg/L
potassium (K)	784	mg/L
**Inorganic trace constituents**		
orthophosphate	0.97	mg/L
fluoride	3.7	mg/L
**Elements (metals and semimetals)**		
barium (Ba)	30.3	µg/L
silicon (calculated as SiO_2_)	14.9	mg/L
strontium (Sr)	161.2	µg/L

**Table 4:** Chemical analysis of permeate in the case of pore wetting for synthetic brine feed with antiscalant.

Physical Parameters	Results	Unit
pH value	7.47	
electrical conductivity	560	µs/cm
**Chemical parameters**		
bicarbonate	33.4	mg/L
calcium (Ca)	<1	mg/L
magnesium (Mg)	1.7	mg/L
nitrate	17.3	mg/L
nitrite	0.038	mg/L
chloride (${\mathrm{Cl}}^{-}$)	116	mg/L
sulfate	53.7	mg/L
iron (Fe)	0.03	mg/L
manganese (Mn)	0.01	mg/L
aluminum (Al)	0.05	mg/L
sodium (Na)	117	mg/L
potassium (K)	2.8	mg/L
**Inorganic trace constituents**		
orthophosphate	<0.02	mg/L
fluoride	0.28	mg/L
**Elements (metals and semimetals)**		
barium (Ba)	<20	µg/L
silicon (calculated as SiO_2_)	1.4	mg/L
strontium (Sr)	20.8	µg/L

**Table 5:** Summary of the results obtained for various feed types.

Parameters	Linz Water	NaCl Solution	Feed without Antiscalant	Feed with Antiscalant	Units
Permeate flux reduction	0	0	44%	60%	-
Initial permeate electrical conductivity	2.8	6.76	10.0	14.7	µS/cm
Final permeate electrical conductivity	2	35.4	4.7	3.7	µS/cm
Initial brine electrical conductivity	534	10,000	10,100	9470	µS/cm
Final brine electrical conductivity	933	31,000	86,600	47,500	µS/cm
Recovery	81%	70%	91%	88%	-
Operational time	65	45	192	167	h

**Figure A1:**
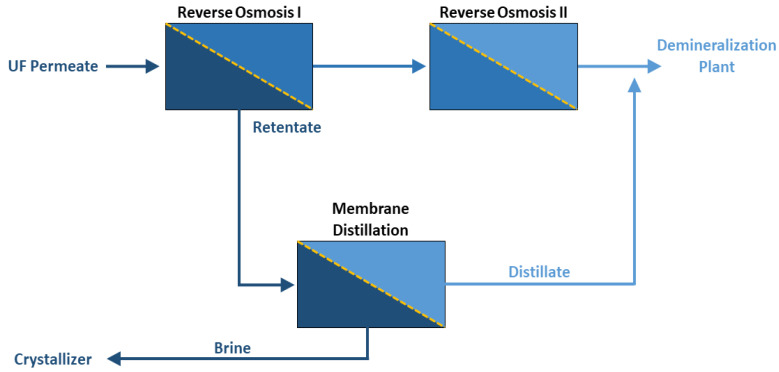
Water desalination by an RO + VMD integrated process. The schematic diagram shows the hybrid reverse-osmosis membrane-distillation process for maximizing the system recovery rate and minimizing the environmental impact of brine waste when a VMD unit is added downstream of the RO unit for further recovery.

**Figure A2:**
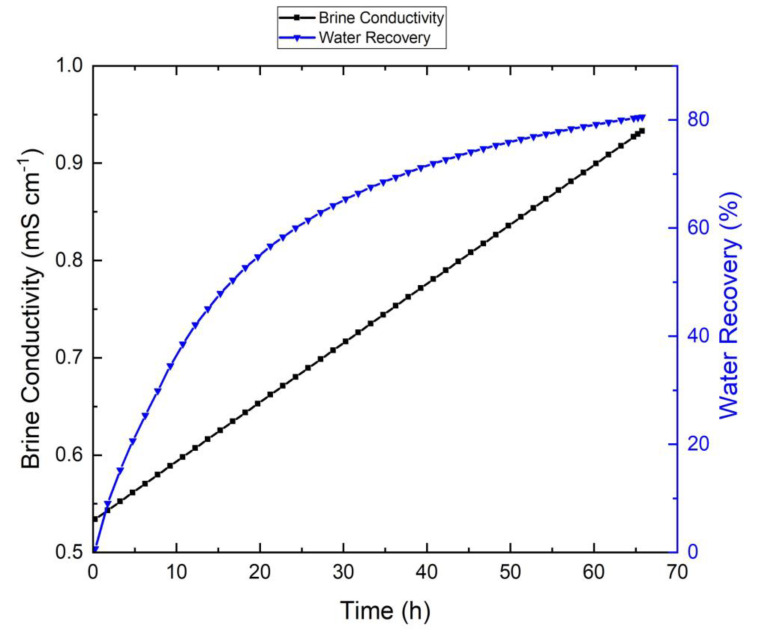
Brine conductivity and water recovery as functions of time for the system with tap water as feed.

**Figure A3:**
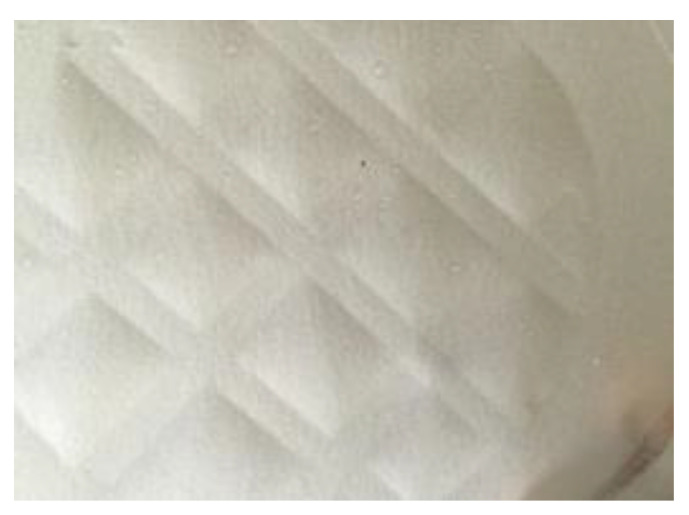
Membrane surface after the NaCl experiment.

**Figure A4:**
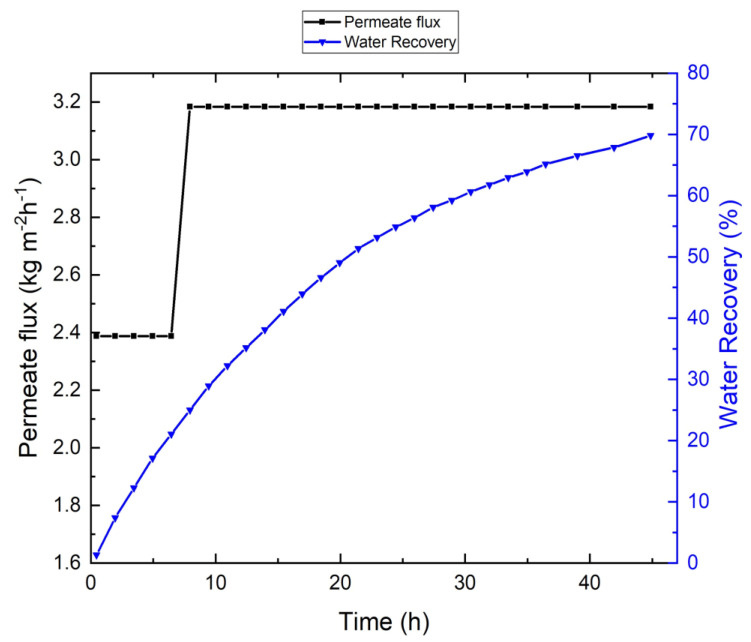
Permeate flux and water recovery as functions of time when using an aqueous NaCl solution as feed.

**Figure A5:**
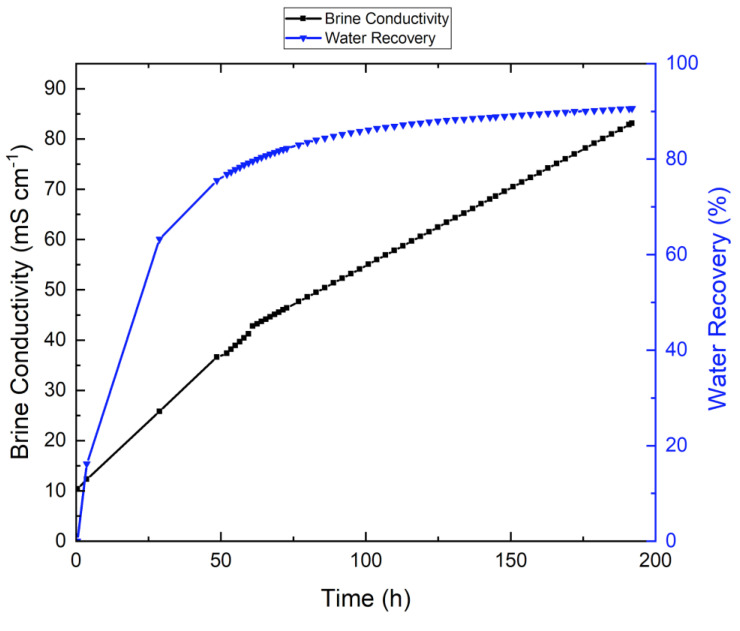
Brine conductivity and water recovery as functions of time for the synthetic brine feed system without antiscalant.

**Figure A6:**
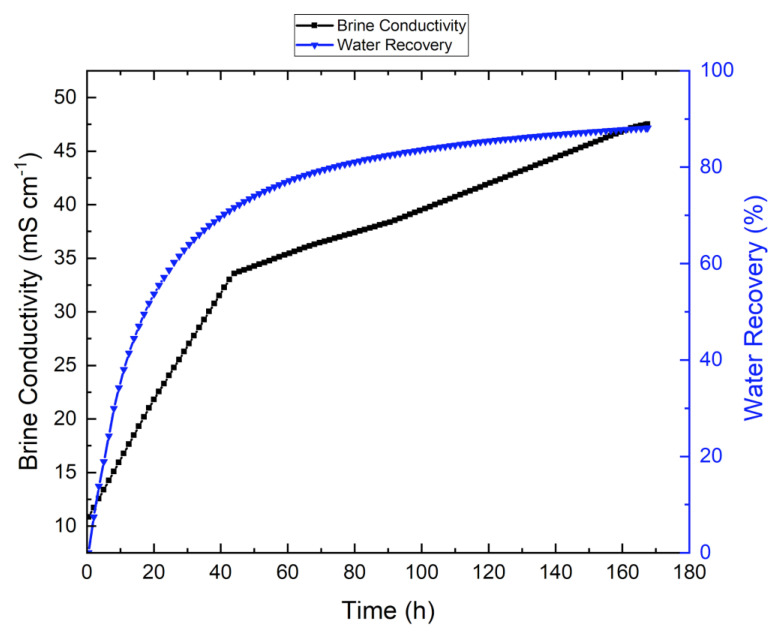
Brine conductivity and water recovery as functions of time for the synthetic brine feed system with antiscalant.

**Figure A7:**
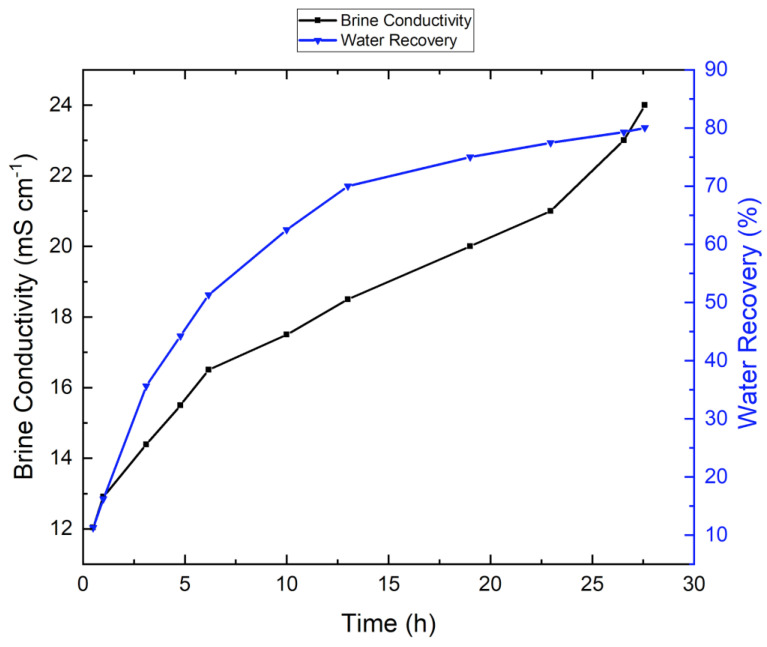
Effect of forced (i.e., high-pressure) wetting on system performance.

**Figure A8:**
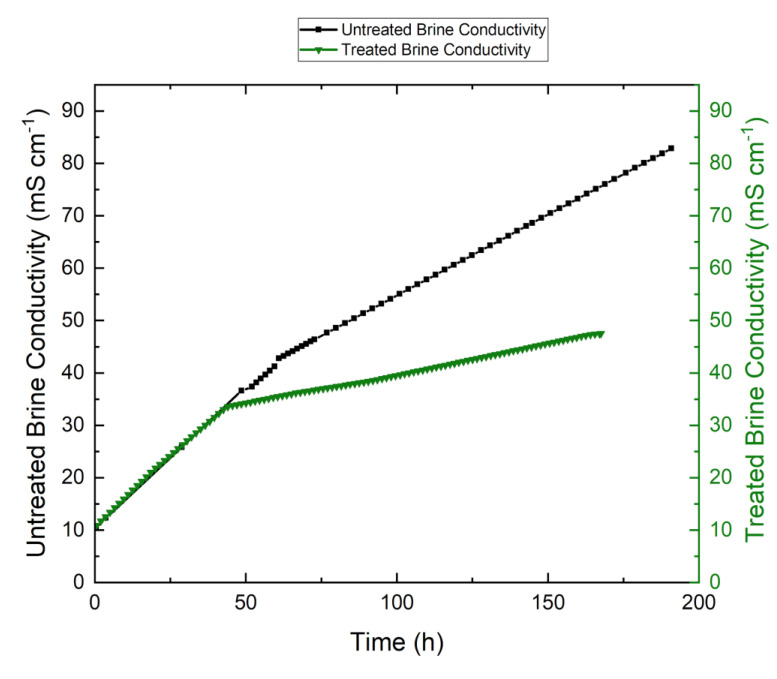
Effect of scaling on system performance.

**Figure A9:**
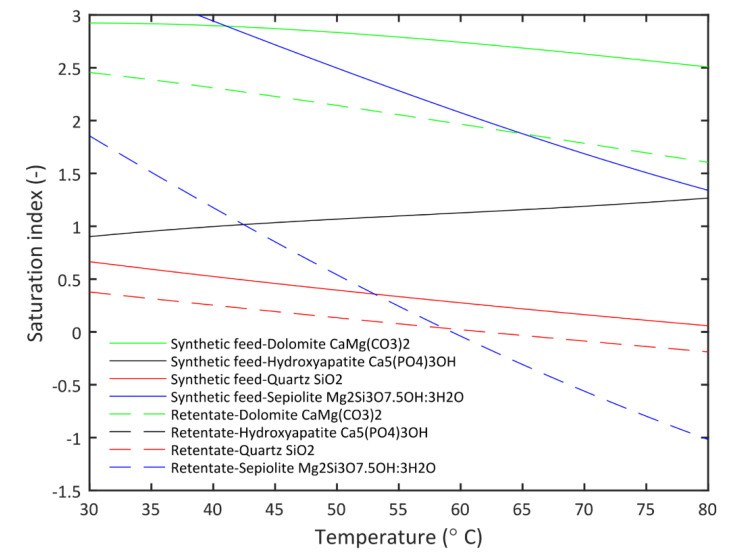
Aqueous speciation equilibrium of synthetic RO feed and retentate. Solid lines indicate the saturation indices of secondary precipitated minerals in the synthetic RO feed, while dashed lines indicate the corresponding saturation indices for the retentate. Modeled using PHREEQC software. At low temperatures, silica minerals typically form colloids rather than precipitates. Dolomite requires temperatures above 100 °C for precipitation activation.

## Nomenclature

f	Concentration factor
K_SP_	Solubility product (molar)
LEP	Liquid entry pressure (mbar)
MD	Membrane distillation
$P_{\left(v,fw\right)}$	Freshwater vapor pressure (mbar)
$P_{\left(v,sw\right)}$	Saline water vapor pressure (mbar)
PP	Polypropylene
PTFE	Polytetrafluoroethylene
RO	Reverse osmosis
$S_{A}$	Absolute salinity (g/kg)
T	Absolute temperature (K)
$M_{C}$	Feed mass under the membrane in the cell
$M_{F}$	Sum of $M_{C}$ and the mass of liquid dispensed from the feed tank
VMD	Vacuum Membrane Distillation
WR	Water recovery

## Appendix A

We carried out experimental lab tests with different feeds under various operating conditions to provide the preliminary settings for MD pilot plant tests developed by VA TECH WABAG GmbH at ESSAR ETP in India. The application of this plant is in a refinery where VA TECH WABAG has installed a wastewater desalination process that comprises flocculation, dual-media filtration, ultrafiltration, and two passes of RO. The total feed flux of the plant is about 500 m^3^/h, and that of the disposal RO brine (i.e., feed to VMD) is up to 69 m^3^/h Pilot plant softening and sand filtration units are installed to pretreat the feed for the MD. At the end of the process, the combined RO and VMD permeates are fed to a demineralization plant for boiler makeup water (Figure A1).

We first prepared 200 L of synthetic RO brine and, for each experiment, we diluted the required amount at a ratio of 1:4. We achieved the estimated feed amount of 1000 L. After gaining information on the permeate flux in initial experiments, we estimated that the volume required for a second feed would be 50 L.

The retentate electrical conductivity for the system with Linz tap water as feed increased linearly from 0.531 to 0.933 mS/cm (Figure A2).

In order to test a simpler system than that with synthetic RO brine, we used an aqueous mono-salt sodium chloride (NaCl) solution of 5.25 g/L with the same initial electrical conductivity as the model water without the antiscalant (10mS/cm). The experiment took 45 h until a recovery ratio of 70%, corresponding to a solution concentration of 17.40 g/L, was reached. Feed temperature and vacuum pressure were kept constant at 75 °C and 30 mbar during the experiment.

Figure A3 and Figure A4 show the surface of the membrane after the experiment and the system performance for the aqueous NaCl solution, respectively. The results showed a lower permeate flux of 3.18 kg m^−2^ h^−1^ compared to the system using Linz tap water feed, and no decrease in flux was detected (<1%).
